# Regulation of prokineticin 2 expression by light and the circadian clock

**DOI:** 10.1186/1471-2202-6-17

**Published:** 2005-03-11

**Authors:** Michelle Y Cheng, Eric L Bittman, Samer Hattar, Qun-Yong Zhou

**Affiliations:** 1 Department of Pharmacology, University of California, Irvine, CA, USA; 2 Department of Biology, University of Massachusetts, Amherst, MA, USA; 3 Departments of Biology and Neuroscience, Johns Hopkins University, Baltimore, MD, USA

## Abstract

**Background:**

The suprachiasmatic nucleus (SCN) contains the master circadian clock that regulates daily rhythms of many physiological and behavioural processes in mammals. Previously we have shown that prokineticin 2 (*PK2*) is a clock-controlled gene that may function as a critical SCN output molecule responsible for circadian locomotor rhythms. As light is the principal zeitgeber that entrains the circadian oscillator, and *PK2 *expression is responsive to nocturnal light pulses, we further investigated the effects of light on the molecular rhythm of *PK2 *in the SCN. In particular, we examined how *PK2 *responds to shifts of light/dark cycles and changes in photoperiod. We also investigated which photoreceptors are responsible for the light-induced *PK2 *expression in the SCN. To determine whether light requires an intact functional circadian pacemaker to regulate *PK2*, we examined *PK2 *expression in cryptochrome1,2-deficient (*Cry1-/-Cry2-/-*) mice that lack functional circadian clock under normal light/dark cycles and constant darkness.

**Results:**

Upon abrupt shifts of the light/dark cycle, *PK2 *expression exhibits transients in response to phase advances but rapidly entrains to phase delays. Photoperiod studies indicate that *PK2 *responds differentially to changes in light period. Although the phase of *PK2 *expression expands as the light period increases, decreasing light period does not further condense the phase of *PK2 *expression. Genetic knockout studies revealed that functional melanopsin and rod-cone photoreceptive systems are required for the light-inducibility of *PK2*. In *Cry1-/-Cry2-/- *mice that lack a functional circadian clock, a low amplitude *PK2 *rhythm is detected under light/dark conditions, but not in constant darkness. This suggests that light can directly regulate *PK2 *expression in the SCN.

**Conclusion:**

These data demonstrate that the molecular rhythm of *PK2 *in the SCN is regulated by both the circadian clock and light. *PK2 *is predominantly controlled by the endogenous circadian clock, while light plays a modulatory role. The *Cry1-/-Cry2-/- *mice studies reveal a light-driven *PK2 *rhythm, indicating that light can induce *PK2 *expression independent of the circadian oscillator. The light inducibility of *PK2 *suggests that in addition to its role in clock-driven rhythms of locomotor behaviour, *PK2 *may also participate in the photic entrainment of circadian locomotor rhythms.

## Background

Light is the principal zeitgeber that entrains circadian rhythms of physiology and behaviour [1,2]. The major light input pathway to the suprachiasmatic nucleus (SCN) is the retinohypothalamic tract [3], which arises from a population of retinal ganglion cells [4]. Recent studies have demonstrated that melanopsin-containing retinal ganglion cells, rods, and cones all convey photic information to the SCN, and mice lacking these photoreceptive systems cannot be entrained by light [5-11]. Excellent progress has been made in the understanding of circadian photic entrainment [12-15]. This includes light-induced transcriptional activation of core clock genes in the SCN, such as *Per1 *and *Per2*, as well as immediate-early gene *c-fos*. Exposure to light pulses at night induces expression of these genes in the SCN, and this light induction mechanism has been suggested as a critical pathway for the resetting of circadian clock in response to changes in light/dark conditions [16-19]. Intercellular signalling mechanisms between SCN neurons are also important in circadian photic entrainment, as mice with mutation in a neuropeptide receptor for VIP (Vasoactive Intestinal Peptide) and PACAP (Pituitary Adenylate Cyclase Activating Peptide) are unable to sustain normal circadian behaviour and exhibit loss of sensitivity to light [20].

In addition to the effect of light on circadian entrainment, light also has a direct effect on physiology and behaviour, generally termed as "masking" [21,22]. For instance, light pulses given at night acutely suppress the locomotor behaviour of nocturnal rodents [21,22], and this can occur without functional clockwork [23-27]. Masking may account for the maintenance under normal light/dark conditions of wheel-running rhythms in cryptochrome-deficient (*Cry1-/-Cry2-/-*) mice, which are behaviourally arrhythmic under constant darkness. The contribution of masking to normal locomotor activity rhythms is unclear, as is the participation of the SCN in masking effects of light. Vitaterna et al (1999) first observed a light-driven *Per2 *rhythm in the SCN in *Cry1-/-Cry2-/- *mice, and have suggested that the light-driven molecular rhythm in the SCN may be related to the preservation of their locomotor rhythm [25].

We previously found that prokineticin 2 (*PK2*) is a first order clock-controlled gene, whose expression in the SCN is regulated by CLOCK and BMAL1 acting on the E-boxes in the gene's promoter [28]. We have also demonstrated that PK2 may function as a SCN output molecule that transmits circadian locomotor rhythm via activation of a G protein-coupled receptor [28,29]. Interestingly, we also observed that *PK2 *expression is rapidly induced by light pulses administered at night [28], a characteristic that is usually seen with core clockwork genes but not clock-controlled genes. Here we further investigated the light regulation of the rhythm of *PK2 *expression in the SCN. In particular, we investigated the photoreceptive mechanisms responsible for the light-induced *PK2 *expression in the SCN. Utilizing *Cry1-/-Cry2-/- *mice, we also determined whether light can drive *PK2 *expression in the SCN independent of a functional circadian clock.

## Results

### *PK2 *responds differentially to the delay and advance of light/dark cycles

**Figure 1:**
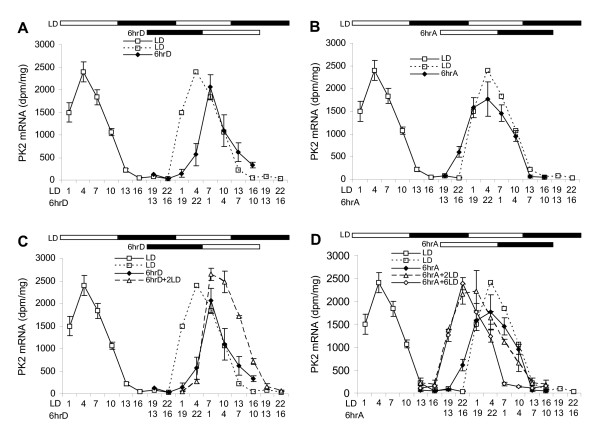
**Temporal profiles of *PK2 *mRNA in the SCN in response to abrupt shifts of light/dark cycles. **Animals were entrained to 12L:12D (LD) and subjected to either 6-hour delay of light/dark cycles (6hrD), 6-hour advance (6hrA), 6-hour delay followed by adaptation of 2 additional LD (6hrD+2LD), or 6-hour advance followed by adaptation of 2 additional LD (6hrA+2LD) or 6 additional LD (6hrA+6LD). Open and filled horizontal bars indicate light and dark periods, respectively. The LD data is doubled plotted as dashed line (open square) in all graphs. The zeitgeber time (ZT) on the x-axis reflects the timescale for LD, 6hrD or 6hrA. Please note that the additional LD adaptation groups use the same timescale as the 6hrD or 6hrA. (A) Temporal profiles of *PK2 *mRNA under 6hrD and LD. Note that *PK2 *mRNA responds quickly to the 6hrD shift. (B) Temporal profiles of *PK2 *mRNA rhythm under 6hrA and LD. Note that *PK2 *mRNA did not adjust to the 6hrA shift. (C) Temporal profiles of *PK2 *mRNA rhythm under 6hrD, 6hrD+2LD and LD to indicate adaptation of *PK2 *rhythm under 6hrD. Note that *PK2 *rhythm is stably entrained to 6hrD after two days. (D) Temporal profiles of *PK2 *mRNA rhythm under 6hrA, 6hrA+2LD, 6hrA+6LD and LD to illustrate adaptation of *PK2 *rhythm under 6hrA. Note that *PK2 *rhythm did not stably entrained to 6hrA until after 6 days. Each value is the mean ± SEM of 3 animals.

We first examined the effects of abrupt shifts of light/dark cycles on *PK2 *mRNA rhythm in the SCN. Animals were first entrained for two weeks under 12 hour light: 12 hour dark (LD), then subjected to either a 6 hour delay (6hrD) shift or 6 hour advance (6hrA) shift of light/dark cycles. We measured *PK2 *mRNA in the SCN of these animals to examine how quickly the *PK2 *mRNA rhythm re-entrains to the shifted light/dark cycles. Under LD, *PK2 *mRNA peaks during the day and remains low or undetectable during the night. During the first cycle of the delayed shift (6hrD), the *PK2 *mRNA rhythm responds quickly: the rising phase of *PK2 *expression adjusts rapidly to the delayed light/dark cycles, while the falling phase still resembles that of the unshifted light/dark cycles (Figure 1A). In contrast, the *PK2 *mRNA rhythm responds very little to a 6 hour advance shift (6hrA). During the first cycle of the advance shift, the *PK2 *oscillation pattern remains similar to that of the unshifted LD (Figure 1B). These changes in *PK2 *expression during 6hrD or 6hrA shift indicate that the endogenous circadian clock exerts dominant control over the *PK2 *rhythm, as *PK2 *expression cannot respond immediately and completely to the shifts of light/dark cycles.

As it normally takes about 1–2 days for locomotor rhythms to stably entrain to phase delays and about 5–6 days to entrain to phase advances [30,31], we next examined the timecourse of shifts of the *PK2 *rhythm to 6 hour phase advances and delays. Consistent with the animal's locomotor behaviour, the *PK2 *mRNA rhythm reaches stable phase within 2 days of 6hrD shift (Figure 1C). In contrast, only the rise of *PK2 *reaches stable phase within 2 days of 6hrA shift, while the fall of *PK2 *takes longer (Figure 1D). Thus, we further examined whether the *PK2 *rhythm is stably entrained after 6 days of 6hrA shift. As expected, the *PK2 *rhythm is completely entrained to 6hrA shift after 6 days (Figure 1D). Together, the differential responses of *PK2 *rhythm to a 6hrD or 6hrA shift indicate that the endogenous circadian clock predominantly controls *PK2 *rhythm, as circadian oscillators typically show rapid phase delays but advance with transients [31,32]. The entrainment patterns of *PK2 *during phase shifts are consistent with behavioural studies in animals and human subjects [30,31].

### *PK2 *rhythm is entrained by different photoperiods

**Figure 2:**
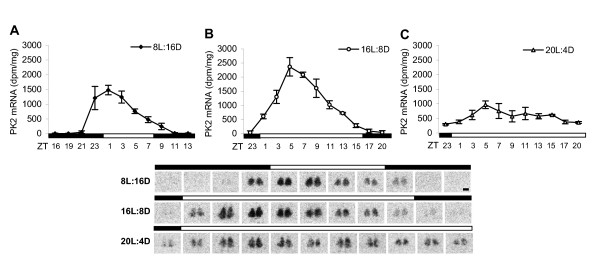
**Effects of photoperiods on *PK2 *mRNA rhythm in the SCN. **Temporal profiles of *PK2 *rhythm under 8L:16D (A), 16L:8D (B) and 20L:4D (C). Open and filled bars indicate light and dark periods, respectively. The zeitgeber time (ZT) on the x-axis reflects the timescale for each photoperiod. Each value represents the mean ± SEM of 3–4 animals. One-way ANOVA indicated that peak levels of all groups are significantly different from each other, p < 0.05–0.001 using Bonferroni's post-hoc test. Autoradiographic images show representative mRNA expression of *PK2 *during these photoperiods.

**Figure 3:**
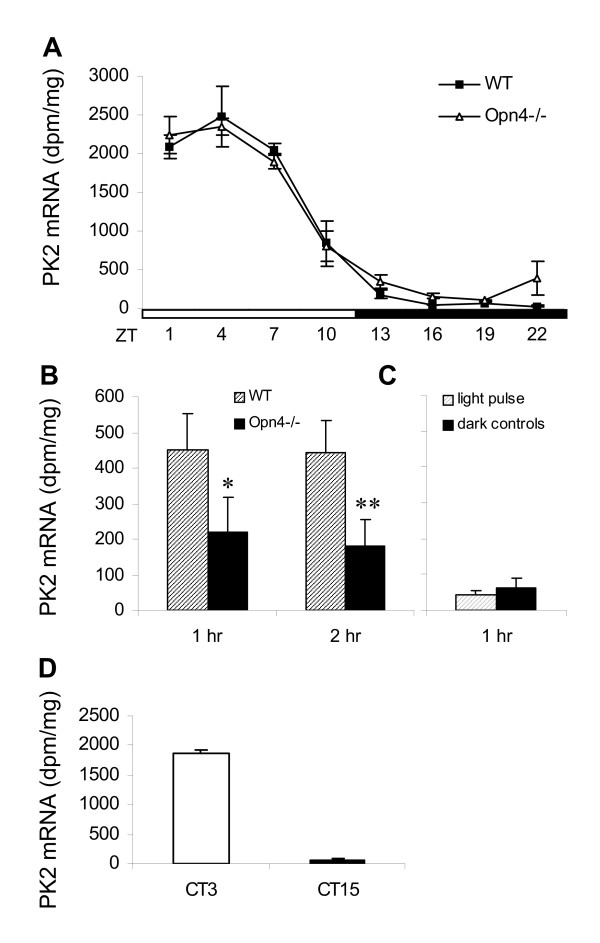
***PK2 *mRNA rhythm in the SCN of melanopsin-deficient (*Opn4-/-*) mice and triple knockout mice (*Opn4-/- Gnat1-/- Cnga3-/- *mice). **(A) Temporal profiles of *PK2 *mRNA rhythm in wildtype (filled squares) and *Opn4-/- *mice (open triangles) under LD. Open and filled bars indicate light and dark periods, respectively. Each value represents the mean ± SEM of 3–4 animals. Two-way ANOVA indicated that there is no significant difference between genotypes. (B) Light pulse-induced *PK2 *mRNA expression in wildtype (shaded bars) and *Opn4-/- *mice (filled bars). *PK2 *mRNA was measured one and two hours after brief light pulse at ZT14. Each value represents the mean ± SEM of 6–8 animals. *p < 0.05, **p < 0.01, Student's t-test. (C) Light-pulse induced *PK2 *mRNA expression in triple knockout mice that lack melanopsin, rod and cone photoreceptive system (*Opn4-/- Gnat1-/- Cnga3-/- *mice). Dark controls received no light pulse. Each value represents the mean ± SEM of 3 animals. (D) *PK2 *mRNA expression in triple knockout mice at circadian time (CT) 3 and 15.

We next examined the effect of photoperiod on the *PK2 *molecular rhythm in the SCN. *PK2 *mRNA was measured in the SCN of mice entrained under different photoperiods: 8 hour light: 16 hour dark (8L:16D), 16 hour light: 8 hour dark (16L:8D), or 20 hour light: 4 hour dark (20L:4D). During 12L:12D, *PK2 *mRNA is highly expressed during the 12 hour light phase with peak level at ZT4 (Figure 1A, Figure 3A). Under 16L:8D, *PK2 *mRNA expands to the entire 16 hour light phase and is essentially undetectable during the 8 hour dark period (Figure 2B). However, the expression of *PK2 *mRNA is not confined to the light phase of the shorter photoperiod (8L:16D), as *PK2 *mRNA rises before lights on and persists after lights off (Figure 2A). The temporal profile of *PK2 *mRNA under this short photoperiod (8L:16D) is very similar to that observed under 12L:12D (Figure 1A, Figure 3A) or constant darkness (2DD) [28]. Thus, although light can induce *PK2 *mRNA and expand the duration of *PK2 *expression, the phase angle of *PK2 *expression is determined by the circadian clock, and its duration cannot be further compressed under shorter photoperiods. Interestingly, the peak of *PK2 *mRNA expression was significantly higher in long days (16L:8D) than in shorter days (8L:16D) (Figure 2A–B), further indicate the enhancing effect of light on *PK2 *expression. However, a significant reduction in the *PK2 *peak level is observed under a very long photoperiod (20L: 4D) (Figure 2C). We also noticed that under 20L:4D, *PK2 *mRNA is further expanded and becomes detectable even in dark phase (Figure 2C). Under this long photoperiod (20L:4D), the difference between the peak and basal level of *PK2 *is only about 4 fold (Figure 2C). As it has been reported that the rhythms of *mPer1 *and *mPer2 *mRNAs in the SCN are also entrained with different phase angles under a variety of photoperiods [33-35], we have also examined *Per1 *and *Per2 *rhythm in our photoperiod studies (see Additional file 1). The *Per1 *and *Per2 *rhythm we observed under these photoperiods are consistent with previous findings [35]. Taken together, these results indicate that changes in photoperiod alter *PK2 *rhythm in the SCN, and the amplitudes of *PK2 *mRNA oscillation are greatly reduced in very long photoperiods.

### Light inducibility of *PK2 *is eliminated in mice that lack melanopsin, rod and cone phototransduction system (*Opn4-/-, Gnat1-/- Cnga3-/- *mice)

As melanopsin has been implicated in circadian photoreception [5-11], we examined whether the *PK2 *molecular rhythm is normally entrained in melanopsin-deficient (*Opn4-/-*) mice. Figure 3 shows that the oscillation profile of *PK2 *in the SCN of *Opn4-/- *mice is essentially identical to that observed in the wild type mice under LD. This normal temporal profile of *PK2 *mRNA corresponds with the normal locomotor rhythm of *Opn4-/- *mice under light/dark conditions [7,8]. As *Opn4-/- *mice display attenuated phase resetting in response to light pulses and exhibit impaired light masking responses to bright light [36], we also examined whether light inducibility of *PK2 *is blunted in *Opn4-/- *mice. Figure 3B shows that light pulse-induced *PK2 *in the SCN of *Opn4-/- *mice was significantly reduced by about 50% and 60%, one and two hours after the light pulse, respectively.

The *Opn4-/- *light pulse studies show that a residual *PK2 *expression is still present after a light pulse, suggesting that without melanopsin, other phototransduction system can still transmit light information to induce *PK2 *expression. Thus, we decided to examine the light inducibility of *PK2 *in triple knockout mice lacking melanopsin, rod and cone phototransduction systems (*Opn4-/- Gnat1-/- Cnga3-/- *mice), as these animals free run under light dark conditions (LD) and lack masking responses to light [10]. Figure 3C shows that the light pulse-induced *PK2 *in the SCN is completely eliminated in these triple knockout mice, consistent with their malfunctioning photoentrainment systems and their lack of masking responses to light [10]. In addition, we also observed that *PK2 *mRNA followed the free-running locomotor rhythms in these triple knockout mice (Figure 3D), with high levels of *PK2 *during the inactive phase (CT3) and low levels during active phase (CT15). Together, these results suggest that melanopsin contributes to the light inducibility of *PK2*, and intact melanopsin with functional rod/cone phototransduction systems are required for the light inducibility of *PK2*.

### A low amplitude *PK2 *rhythm is preserved in cryptochrome-deficient (*Cry1-/-Cry2-/-*) mice under light/dark conditions

**Figure 4:**
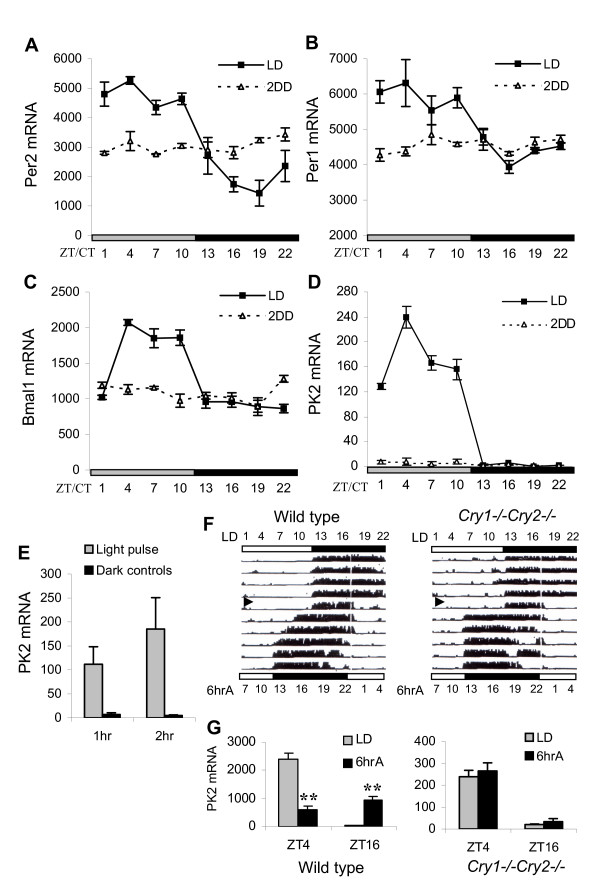
**Light-driven molecular rhythms in the SCN of *Cry1-/-Cry2-/- *mice. **Temporal mRNA profiles of *Per2 *(A), *Per1 *(B), *Bmal1 *(C) and *PK2 *(D) in *Cry1-/-Cry2-/- *mice under 12L:12D (LD) or two days constant darkness (2DD). Each value represents the mean ± SEM of 3–4 animals. Two-way ANOVA with Bonferroni's posthoc analysis was used to test for significant interactions between expression across time of sampling and under different lighting conditions (LD vs 2DD). p < 0.0001 (Per2), p < 0.002 (Per1), p < 0.0001 (Bmal1) and p < 0.0001 (*PK2*). (E) Light pulse-induced *PK2 *mRNA in *Cry1-/-Cry2-/- *mice. *PK2 *mRNA was measured one and two hours after brief light pulse at ZT14 (shaded bar). Black bar represents dark controls that did not receive light pulse. Each value represents the mean ± SEM of 5–6 animals. Two-way ANOVA indicates a significant difference in *PK2 *expression between light and dark treatment (p < 0.05), however, the *PK2 *induction is not significantly different between the two timepoints (1 hr vs 2 hr). (F) Locomotor behavioural rhythms of wild type (left) and *Cry1-/-Cry2-/- *mice (right) in response to 6 hour advance of light/dark cycle. Open and filled bars indicate light and dark periods, respectively. Black arrow indicates the day of 6 hour advance shift (6hrA). Numbers above and below the actograms represent timescale in zeitgeber time (ZT) for LD and 6hrA. (G) Rapid adjustment of *PK2 *rhythm in *Cry1-/-Cry2-/- *mice to 6 hour advance (6hrA). *PK2 *mRNA was quantitated in the SCN of wildtype and *Cry1-/-Cry2-/- *mice under LD (shaded) or 6hrA (black). Each value represents the mean ± SEM of 3–4 animals. Three-way ANOVA with Bonferroni's post hoc analysis indicates a significant interaction between light/dark cycle (LD vs 6hrA), timepoint (ZT4 vs ZT16) and genotype (wildtype vs *Cry1-/-Cry2-/- *mice), p < 0.001. Two-way ANOVA with Bonferroni's post hoc analysis show that there is significant difference in wildtype *PK2 *expression level between LD and 6hrA, (ZT4, ***p < 0.001; ZT16, **p < 0.01), but not in *Cry1-/-Cry2-/- *mice (ZT4, p = 1.000; ZT16, p = 1.000).

Previous studies have shown that the light-regulated *Per2 *rhythm is maintained in the SCN of cryptochrome-deficient (*Cry1-/-Cry2-/-*) mice that lack functional circadian clock [25,37]. In order to determine whether the regulation of *PK2*, *Per1*, *Per2 *and *Bmal1 *expression by light requires an intact circadian pacemaker, we systematically assessed the temporal mRNA profiles of clockwork genes in *Cry1-/-Cry2-/- *mice under both light/dark (LD) and constant dark (DD) conditions. Figure 4 shows that the molecular rhythm of *Per2 *remained largely intact in *Cry1-/-Cry2-/- *mice entrained under12L:12D, with levels about 4-fold higher during the light phase than the dark phase. This amplitude of the *Per2 *oscillation profile was similar to that observed in wild type mice [18,38]. A low amplitude *Per1 *rhythm in *Cry1-/-Cry2-/ *mice was also apparent under LD, but not DD (Figure 4B). We further detected a light-driven *Bmal1 *rhythm in the SCN of *Cry1-/-Cry2-/- *mice under LD, but not DD (Figure 4C). Interestingly, this *Bmal1 *rhythm in *Cry1-/-Cry2-/- *mice peaked during light phase, opposite from the *Bmal1 *rhythm in wild type mice and in phase with *Per1 *[39,40]. As it has been suggested that PER2 can positively regulate *Bmal1 *expression via inhibition of the orphan nuclear receptor REV-ERBα [41,42], it is possible that this *Bmal1 *rhythm is secondary to the light-driven *Per2 *rhythm. Further studies are required to clarify this observation.

We also examined the molecular rhythm of *PK2 *in *Cry1-/-Cry2-/- *mice. Figure 4D shows that *PK2 *mRNA rhythm in the SCN of *Cry1-/-Cry2-/- *mice was apparent under LD, with the presence of a low level *PK2 *during light phase and absence of *PK2 *during dark phase (see Additional file 2). Similar to wild type mice, the peak level of this low amplitude *PK2 *rhythm was around ZT4, although its peak was only about 8% of that observed in wild type mice (Figure. 4D, Figure 1A, Figure 3A). No *PK2 *rhythm was evident when *Cry1-/-Cry2-/- *mice were placed under DD (Figure 4D). Furthermore, the inducibility of *PK2 *to nocturnal light pulses is also maintained in *Cry1-/-Cry2-/- *mice. *PK2 *mRNA increased one and two hours after a brief light pulse at ZT14 (Figure 4E). Nevertheless, light-induced *PK2 *was still detected in *Per1,2,3-/- *mice and *Clk-/- *mice that lack functional circadian clock (Cheng, Weaver & Zhou, unpublished observations). As *PK2 *remains responsive to light in these clock mutant mice that lack functional circadian clock, it is likely that the low amplitude *PK2 *rhythm in *Cry1-/-Cry2-/- *mice under LD is directly driven by light.

In order to test whether this light-driven *PK2 *rhythm may be related to the maintenance of behavioural rhythms observed in *Cry1-/-Cry2-/- *mice under LD, we studied the responses of *Cry1-/-Cry2-/- *mice to a 6 hour advance of lighting schedule. In contrast to the transients of entrainment of locomotor rhythms in wild type mice (which takes about 4-5 days to re-entrain to phase advance), the locomotor activity of *Cry1-/-Cry2-/- *mice adjusted rapidly to 6 hr advance (Figure 4F). Such a rapid response is characteristic of masking. A correlative rapid adjustment of *PK2 *was also observed in the SCN of *Cry1-/-Cry2-/- *mice (Figure 4G). As *Cry1-/-Cry2-/- *mice lack functional circadian clock and their locomotor behaviour and *PK2 *expression patterns are completely light driven, our results suggest that this low amplitude, light-driven rhythm of *PK2 *may contribute to or underlie the masking of locomotor behaviour in these animals.

## Discussion

Our studies indicate that the molecular rhythm of *PK2 *in the SCN is predominantly controlled by the circadian clock, with light playing a modulatory role. Abrupt shifts of light/dark cycles significantly altered the phase of the *PK2 *rhythm. While *PK2 *expression re-entrained rapidly to phase delays, it takes several cycles of transients for *PK2 *to be stably entrained to phase advances (Figure 1). The rate of re-entrainment of *PK2 *molecular rhythms to these shifts is consistent with that of behavioural adaptation of animals and human subjects [30,31]. Our photoperiod studies indicate that *PK2 *expression in the SCN responds differentially to changes in photoperiod length (Figure 2). Although increasing light period can induce *PK2 *expression and expand the duration of *PK2 *rhythm (Figure 2B), shortening of the light period does not lead to corresponding reduction of the duration of *PK2 *expression (Figure 2A). It appears that a minimal duration of *PK2 *expression is maintained under short photoperiod (Figure 2A) and constant darkness [28], which further indicate the dominant control of *PK2 *expression by the circadian clock. Interestingly, the amplitude of the *PK2 *oscillation was greatly reduced under very long photoperiod (20L:4D) (Figure 2C). As the amplitude of both *Per1 *and *Per2 *rhythms were also reduced during 20L:4D (see Additional file 1), it is likely that these depressed rhythms of clockwork genes may contribute to the depressed *PK2 *rhythm observed. Whether reduction in the amplitude of expression in any of these genes is related to arrhythmicity in LL deserves further examination.

Our studies with *Cry1-/-Cry2-/- *mice revealed the presence of a light-driven *PK2 *molecular rhythm in the SCN under LD, indicating that light can drive *PK2 *rhythm independent of functional circadian clock. Interestingly, the molecular rhythms of some clockwork genes such as *Per2*, *Per1*, and *Bmal1 *were also partially maintained in the SCN of *Cry1-/-Cry2-/- *mice under LD (Figure 4). Thus, light-driven molecular oscillations of clockwork or clock-controlled output genes exist in the absence of functional circadian clock. Vitaterna et al (1999) first noticed such light-regulated *Per2 *molecular rhythm in the SCN of *Cry1-/-Cry2-/- *mice, and suggested the term of "light-driving" effect [25]. As *Cry1-/-Cry2-/- *mice lack functional circadian clocks and their locomotor behaviour remains rhythmic under LD, but not under DD conditions [24,25], it is likely that these light-driven molecular rhythms may drive the locomotor rhythms in these animals. As we have previously shown that *PK2 *may be a critical output molecule responsible for circadian locomotor rhythms, the presence of this light-driven *PK2 *rhythm in *Cry1-/-Cry2-/- *mice may thus contribute to or underlie masking as well as the free running behavioural rhythms in these animals. It is well established that an intact SCN is necessary for the preservation of free running locomotor rhythms [43]. The role of the SCN in masking of locomotor activity by light is controversial, with similar studies having produced contradictory results [23,44]. Thus, it is possible that there might be common signal molecule(s) that mediate(s) the light-masking and the circadian clock-controlled locomotor behaviour. Construction of *PK2*-deficient mice will be necessary to resolve the exact role of PK2 in the light-driven locomotor rhythms.

The light inducibility of *PK2 *in the SCN is an unusual characteristic for a clock-controlled gene. Our results demonstrate that melanopsin-positive retinal ganglion cells, in conjunction with rods and cones, are responsible for the light-inducibility of *PK2 *(Figure 3). The same photoreceptive system has been shown responsible for the entrainment of locomotor rhythm [5-11]. The light inducibility of *PK2 *may be related to the presence of a putative cyclic AMP response element (CRE) in the promoter of the *PK2 *gene [28]. CRE-dependent activation is critical for light-induced gene expression in the SCN [45-48]. The reduced light inducibility of *PK2 *in mutant mice that lack functional clock may indicate that CRE-dependent pathway and CLK/BMAL1 transcriptional factors may interact in the light-induced *PK2 *expression in the SCN. Accumulative data have implicated the photic regulation of the transcription of clock genes such as *Per1 *and *Per2 *in the entrainment of behavioural rhythms [30,34]. The phase of the core SCN clock gene expression determines the timing of clock-controlled SCN output signals that ultimately regulate physiology and behaviour. Unlike the *Per1 *promoter, whose activation in the SCN shifts rapidly when the LD cycle is advanced [31], *PK2 *exhibits transients during phase advance, more similar to those of *Cry1 *and *Cry2 *[30,31]. This is consistent with the role for PK2 as a clock-controlled gene and thus is downstream from the light-regulated expression of *Per1 *or *Per2*. The presence of E box motifs in the *PK2 *promoter suggests that light-regulated *Per1 *(and perhaps *Per2*) expression can influence *PK2 *expression. However, the light inducibility of *PK2 *indicates that PK2 may have a more direct and central role in entrainment in addition to its putative role as an SCN output signal. In other words, whether PK2 functions completely outside the central circadian loops or partly within them has yet to be determined. It is well established that the activation of glutamate receptor and its downstream actions are critical for the retinohypothalamic inputs of light to the SCN [49]. As receptor for PK2 is highly expressed in the SCN [28] and activation of the PK2 receptor triggers similar signalling pathways as that of glutamate receptors [29], it is possible that the circadian clock and/or light-driven PK2 may feed back to the core circadian loops in the SCN. In addition, PK2 has recently been shown to excite neurons that express PK2 receptor [50], further suggesting that PK2 may activate the firing of SCN neurons, and thus possibly participate in the synchronization of the circadian clock. Thus, the light inducibility of *PK2 *may be relevant to both the phase resetting of the core circadian loops and critical SCN output signals.

## Conclusion

Our studies demonstrate that *PK2 *is predominantly driven by the circadian clock, as *PK2 *expression exhibits circadian transients in response to phase advances. Furthermore, shortening of the light period does not result in corresponding reduction of the phase of *PK2 *rhythm, also consistent with the dominant control from the circadian clock on *PK2 *expression. However, light also modulates *PK2 *rhythm. Nocturnal light pulses can directly induce *PK2 *expression in the SCN. Studies with *Cry1-/-Cry2-/- *mice revealed that light can drive a low amplitude *PK2 *molecular rhythm in the SCN in the absence of functional circadian oscillators. These studies demonstrate that *PK2 *molecular rhythm in the SCN is controlled by dual mechanisms: dominantly by the circadian transcriptional loops but also directly by light. The light inducibility of *PK2 *in the SCN suggest that in addition to PK2's role as a SCN output signal, PK2 may also participate in the photic entrainment of circadian clock and perhaps in masking.

## Methods

### Experiments of light/dark cycle shifts

Male adult C57BL/6 mice (Taconic Farms, New York) were entrained under 12 hour light: 12 hour dark (12L:12D, lights on at 0700 h) cycle for two weeks with food and water available ad libitum. Light phase was either delayed by 6 hours (lights on at 1300 h) or advanced by 6 hours (lights on at 0100 h) and samples were taken every three hours for the 24 hour period (Zeitgeber time, ZT, ZT1-22). To examine *PK2 *expression two days after the shift, animals were placed in two additional light/dark cycles and brain samples were collected. All animal procedures were approved by the Institutional Animal Care and Use Committee and consistent with Federal guidelines. In situ hybridization was used in all studies to examine *PK2 *mRNA expression in the SCN [28]. Antisense and sense riboprobes containing the coding region of mouse *PK2 *(accession number AF487280 1-528 nt), mouse *Per1 *(accession number AF022992 340-761nt), mouse *Per2 *(accession number AF035830 9-489 nt) and mouse *Bmal1 *(accession number AB015203 864-1362 nt) were generated.

### Photoperiod studies

Animals were initially entrained under 12L:12D for one week, followed by placement in different photoperiods (light intensity ~400 lux) for three to four weeks: 8 hour light:16 hour dark (8L:16D, lights on at 0900 h, lights off at 1700 h), 16 hour light: 8 hour dark (16L:8D, lights on at 0500 h, lights off at 2100 h). For the 20 hour light: 4 hour dark (20L:4D, lights on at 0300 h, lights off at 2300 h), animals were first placed in 14L:10D for one week, transferred to 16L:8D for another week, followed by two weeks in 20L:4D. All brain samples were taken every two hours throughout the 24 hour cycle, except the first and the last two time points which were sampled every three hours.

### Studies of melanopsin-deficient mice and mice that lack melanopsin, rods and cones

Wild type and melanopsin-deficient (*Opn4-/-*) mice (on C57BL/6:129 hybrid background) [5] were entrained to 12L:12D and sampled every three hours for the 24 hour period (ZT1-22). For light pulse studies, wild type, *Opn4-/- *mice and triple knockouts (*Opn4-/- Gnat1-/- Cnga3-/- *mice) that lack melanopsin, rod and cone phototransduction systems were used [10]. Animals received a 15 min light pulse (~200 lux) at ZT14 and brains were sampled one or two hours after light pulse. Dark control animals did not receive a light pulse.

### Studies of cryptochrome-deficient (*Cry1-/-Cry2-/-*) mice

Cryptochrome-deficient (*Cry1-/-Cry2-/-*) mice on a C57BL/6:129 hybrid background were kindly provided by Dr. Aziz Sancar (University of North Carolina at Chapel Hill). Wild type and *Cry1-/-Cry2-/- *mice were entrained to 12L:12D and sampled every three hours for the 24 hour period (ZT1-22). A second group of *Cry1-/-Cry2-/- *mice were placed into two days of constant darkness (2DD) (Circadian time, CT, CT1-22). The mRNA levels of *PK2*, *Per2*, *Per1 *and *Bmal1 *were measured in the SCN. For light pulse experiments, *Cry1-/-Cry2-/- *mice received a 15 min light pulse (~400 lux) at ZT14, and sampled one or two hours after light pulse. Dark control *Cry1-/-Cry2-/- *mice did not receive a light pulse. For the shifting experiments, wildtype and *Cry1-/-Cry2-/- *mice were initially entrained under 12L:12D, then subjected to an acute 6 hour advance of lighting schedule. Running-wheel activities of these mice were monitored 10 days before and 10 days after the 6 hour advance shift. The 6 hour phase advance was then repeated and brains were collected at ZT4 and ZT16 on the day of the shift.

## Authors' contributions

ELB sampled the *Cry1-/-Cry2-/- *mice and performed behavior experiments on *Cry1-/-Cry2-/- *mice. SH sampled the melanopsin-deficient mice and triple knockout mice. MYC performed the tissue sectioning, in situ hybridizations and all quantitative analyses. MYC, ELB and QYZ drafted the manuscript. ELB, SH, MYC and QYZ designed the studies. All authors read and approved the final manuscript.

## Supplementary Material

Additional File 1 Effect of different photoperiods on molecular rhythms in the SCN. Temporal profiles of Per1 (a) and Per2 (b) mRNA under 8L:16D, 16L:8D, 20L:4D. Open and filled bars indicate light and dark periods, respectively. The zeitgeber time (ZT) on the x-axis reflects the timescale for each photoperiod. Each value represents the mean ± SEM of 3–4 animals. Click here for file

Additional File 2 PK2 mRNA expression in Cry1-/-Cry2-/- and wildtype mice. Representative autoradiograms of PK2 mRNA in the SCN of Cry1-/-Cry2-/- mice (Cry) and wild type mice (WT) under LD (ZT1-22) are shown (top and bottom row, respectively). Scale bar = 1 mm. Click here for file
