# Supplementary figures and images for: Regulation of prokineticin 2 expression by light and the circadian clock
Source: BMC Neurosci. 2005 Mar 11;6:17. doi: 10.1186/1471-2202-6-17 (PMC555564; doi:10.1186/1471-2202-6-17)

**A**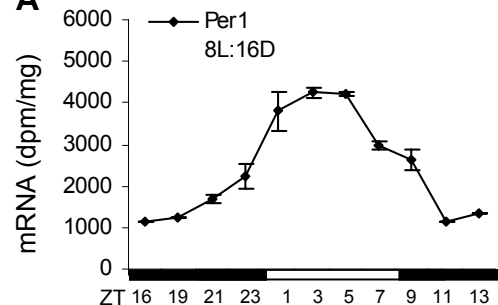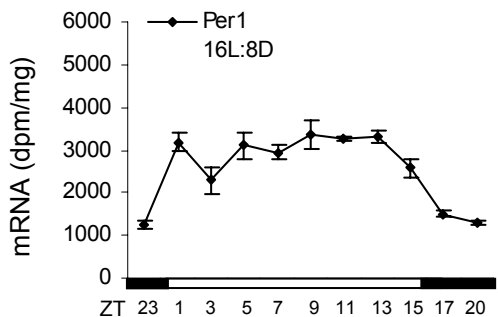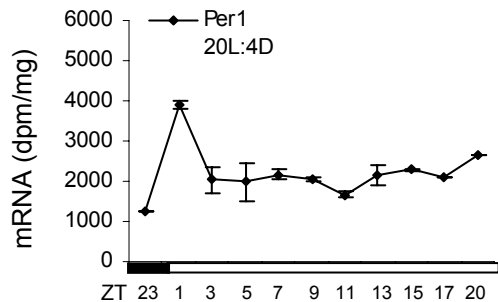**B**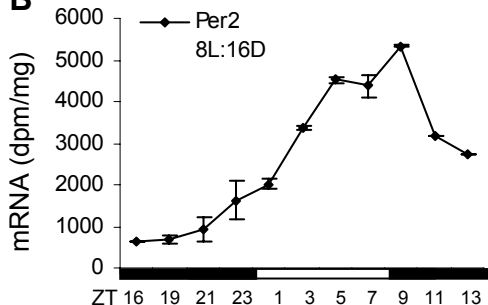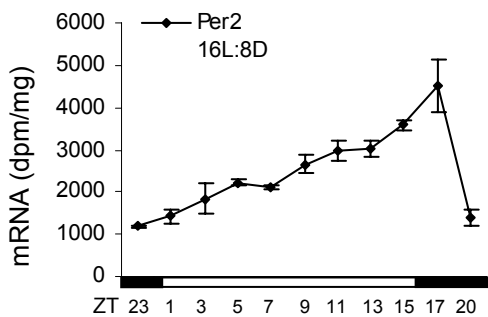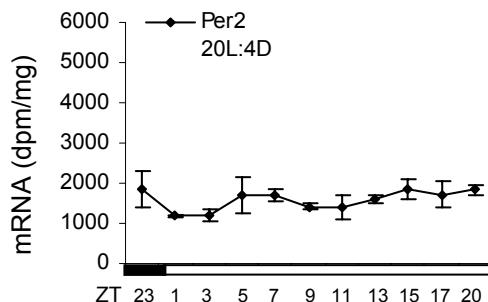

Supplement: Additional File 1 — Effect of different photoperiods on molecular rhythms in the SCN. Temporal profiles of Per1 (a) and Per2 (b) mRNA under 8L:16D, 16L:8D, 20L:4D. Open and filled bars indicate light and dark periods, respectively. The zeitgeber time (ZT) on the x-axis reflects the timescale for each photoperiod. Each value represents the mean ± SEM of 3–4 animals. [file 1471-2202-6-17-S1.pdf]

**ZT1**

**4**

**7**

**10**

**13**

**16**

**19**

**22**

**Cry**

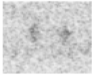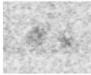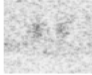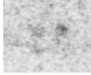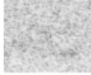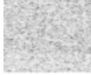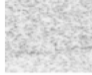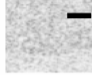

**WT**

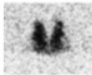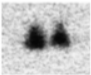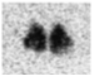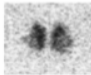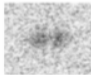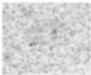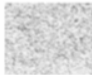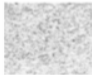

Supplement: Additional File 2 — PK2 mRNA expression in Cry1-/-Cry2-/- and wildtype mice. Representative autoradiograms of PK2 mRNA in the SCN of Cry1-/-Cry2-/- mice (Cry) and wild type mice (WT) under LD (ZT1-22) are shown (top and bottom row, respectively). Scale bar = 1 mm. [file 1471-2202-6-17-S2.pdf]
